# Prenatal opioid exposure and risk of asthma in childhood: a population-based study from Denmark, Norway, and Sweden

**DOI:** 10.3389/fphar.2023.1056192

**Published:** 2023-05-04

**Authors:** Ingvild Odsbu, Marte Handal, Vidar Hjellvik, Sonia Hernandez-Diaz, Helle Kieler, Mette Nørgaard, Svetlana Skurtveit, Buket Öztürk Esen, Milada Mahic

**Affiliations:** ^1^ Department of Mental Disorders, Norwegian Institute of Public Health, Oslo, Norway; ^2^ Centre for Pharmacoepidemiology, Department of Medicine, Karolinska Institutet, Solna, Stockholm, Sweden; ^3^ Department of Chronic Diseases, Norwegian Institute of Public Health, Oslo, Norway; ^4^ Norwegian Centre for Addiction Research (SERAF), University of Oslo, Oslo, Norway; ^5^ Department of Epidemiology, Harvard T. H. Chan School of Public Health, Boston, MA, United States; ^6^ Department of Laboratory Medicine, Karolinska Institutet, Solna, Stockholm, Sweden; ^7^ Department of Clinical Epidemiology, Aarhus University Hospital and Department of Clinical Medicine, Aarhus University, Aarhus, Denmark

**Keywords:** opioids, childhood asthma, pregnancy, prenatal exposure, observational study

## Abstract

**Background:** Opioids may modulate the immune function through opioid receptors on immune cells. Long-term consequences of prenatal opioid exposure on the immune system, such as childhood asthma, are unknown.

**Objectives:** To investigate whether prenatal opioid exposure is associated with the risk of childhood asthma.

**Methods:** Cohort study using linked nationwide registers in Denmark (1996–2015), Norway (2005–2015), and Sweden (2006–2013). Children born by mothers who were chronic opioid analgesics users before pregnancy (*n* = 14,764) or who were receiving opioid maintenance therapy (OMT) before or during pregnancy (*n* = 1,595) were identified based on information from each of the medical birth registers and prescription registers. Long-term opioid analgesics exposed children were compared to short-term exposed or unexposed, whereas OMT exposed children were compared to OMT unexposed. Asthma among children ≥1 years of age was defined as two or more filled prescriptions of antiasthmatic medication within 365 days, or a diagnosis of asthma. Hazard ratios (HRs) were calculated using Cox proportional hazards regression with attained age as the time scale. Inverse probability of treatment weights based on propensity scores were applied to adjust for measured confounders. Individual level data from Norway and Sweden were pooled, whereas individual level data from Denmark were analyzed separately. For the opioid analgesics comparisons, adjusted HRs (aHR) from the combined Norwegian/Swedish data and the Danish data were pooled in a fixed-effects meta-analysis.

**Results:** For the opioid analgesics cohort, no increased risk of asthma was observed in long-term exposed children neither compared with unexposed [aHR = 0.99 (95% CI 0.87-1.12)], nor compared with short-term exposed [aHR = 0.97 (0.86-1.10)]. No increased risk of asthma was observed in OMT exposed compared with OMT unexposed children [Norway/Sweden: aHR = 1.07 (0.60-1.92), Denmark: aHR = 1.25 (0.87-1.81)]. Results from sensitivity analyses, where potential misclassification of the outcome and misclassification of OMT exposure were assessed, as well as starting follow-up at 6 years of age, showed that the estimates of association were generally robust.

**Conclusion:** We found no association between prenatal exposure to opioids and risk of childhood asthma. Results were consistent across two different opioid exposure groups with different confounder distributions.

## Introduction

Asthma is one of the most common chronic diseases among children with high burden for affected individuals as well as for the health care system (Lai et al., 2009; Serebrisky and Wiznia, 2019). If not diagnosed and controlled properly, children with asthma are at higher risk of future morbidity and mortality (Serebrisky and Wiznia, 2019; Caffrey Osvald et al., 2020). The mechanisms behind the development of childhood asthma are largely unknown. Prenatal exposures might affect immune programming during fetal development and trigger immune-mediated diseases, including asthma, later in life (Olin et al., 2018). Asthma is characterized by imbalance of the immune system, overactive Th2 immune response and a downregulated Th1 immune response, and exposures with the ability to skew the immune response towards a Th2 phenotype could potentially increase the risk of asthma development (Holtzman, 2012; Douros et al., 2017). Opioids are found to modulate the immune system through opioid receptors on immune cells and affect the balance of the immune response (Vallejo et al., 2004; Al-Hashimi et al., 2013; Plein and Rittner, 2018). Since opioids pass the placenta (Malek and Mattison, 2011), prenatal exposure to opioids might modulate the immune function of the developing fetus.

Pregnant women use opioid analgesics for pain treatment and the prevalence of use during pregnancy has been reported to be from 2.9% to 5.1% in the Nordic countries (Handal et al., 2011; Stephansson et al., 2011; Faltmarch et al., 2019), and as high as 14.4%–21.6% in the US (Bateman et al., 2014; Desai et al., 2014). Clinical guidelines in the Nordic countries recommend using opioid analgesics for the shortest possible time and at the lowest effective dose if such treatment is indicated during pregnancy (Läkemedelsboken, 2015; Dansk Selskab for Obstetrik og Gynækologi, 2016; Norsk legemiddelbok, 2019). In addition to treatment of pain, pregnant women receive opioids (methadone or buprenorphine) for treatment of opioid use disorder [opioid maintenance treatment (OMT)]. Clinical guidelines recommend that pregnant women already receiving OMT continue their treatment throughout pregnancy, or that OMT is initiated in pregnant women not already receiving such treatment (Handal et al., 2020). Most women receiving OMT are of childbearing age (Gyarmathy et al., 2009). Since 1997 in Denmark, 2005 in Norway and 2006 in Sweden, approximately 800 women were reported to have received OMT drugs during pregnancy (Handal et al., 2020). Over the past decades, an increasing use of opioid analgesics was observed, not only in the general population, but also in the pregnant population (Bjorn et al., 2011; Desai et al., 2014; Muller et al., 2019). The increased use of opioid analgesics could potentially put more individuals at risk of developing opioid use disorder.

For children exposed prenatally to opioids, there seems to be increased risk of adverse birth outcomes and neonatal outcomes such as neonatal abstinence syndrome (Desai et al., 2015; Nørgaard et al., 2015; Wurst et al., 2016; Sujan et al., 2019). In contrast, evidence on long-term consequences of prenatal opioid exposure on immune reactions is scarce. A recent study found no evidence for an association between prenatal opioid exposure and risk of infections in the offspring (Mahic et al., 2020). Some epidemiological studies have investigated the association between prenatal exposure to analgesics and risk of childhood asthma with conflicting results (Källén et al., 2013; Cheelo et al., 2015; Magnus et al., 2016; Fan et al., 2017; Shaheen et al., 2019).

As an increasing number of pregnant women use opioids, more knowledge is needed about the long-term consequences of proposed opioid-induced immunomodulation in the fetus. In this population-based cohort study, we examined the association between prenatal opioid exposure and childhood asthma in live-born children from Denmark, Norway, and Sweden. To minimize any background differences between the groups, we compared children whose mothers had similar indications for opioid use, either long-term opioid use for pain or opioid use disorders. A potential dose-response relationship was also assessed.

## Materials and methods

### Study setting and data sources

Data were retrieved from the national health- and population registers in Denmark, Norway, and Sweden. The Nordic countries have comparable public healthcare systems and population-based registers, and all citizens have unique personal identification numbers which makes it possible to link data sources within a country (Laugesen et al., 2021). The medical birth registers contain information on all deliveries from week 12 (Norway) and week 22 (Denmark and Sweden) of gestation (Langhoff-Roos et al., 2014). The personal identity numbers of the live-born infant and parents are recorded. The prescription registers contain information on all filled prescriptions to patients in ambulatory care (Furu et al., 2010). Data on drugs administered in hospitals are not captured in the prescription registers. Drugs are classified according to the Anatomic Therapeutic Chemical (ATC) classification system (WHO, 2021a). The national patient registers capture hospitalizations and visits in outpatient specialist care (the latter not captured in Denmark). Diagnoses are recorded according to the International Classification of Diseases, 10th revision (ICD-10) (WHO, 1992). The patient registers do not capture visits in primary care. In Norway, data on diagnosis from primary care [ICPC-2, International Classification of Primary Care, 2nd edition (WONCA, 1998)] were retrieved from the Control and Payment of Health Reimbursement (KUHR) database (The Norwegian Directorate of Health, 2022). Data on cause of death (ICD-10) were retrieved from the cause of death registers, and information on emigration (not Sweden) was retrieved from the population registers. Individual level data from Norway and Sweden were pooled, whereas individual level data from Denmark were analyzed separately due to legal restrictions on data sharing between countries.

### Study population

A cohort of children born in Denmark 1997–2015, Norway 2005–2015, or Sweden July 1 2006–2013, and whose mothers used opioids pre-pregnancy (opioid analgesics, illicit opioids or OMT), was identified from the medical birth registers and prescription registers. A total of 1,471,406 children born in Norway/Sweden and 1,207,989 children born in Denmark were eligible for inclusion in the study. There were 14,764 children in the opioid analgesics cohort and 1,595 children in the OMT cohort.

### Pre-pregnancy opioid use

Opioids included in the study were opioid analgesics (ATC code N02A) and opioids used for OMT. OMT drugs included methadone oral solution (ATC code N07BC02) and high-dose buprenorphine tablets (≥2 mg sublingual tablets, ATC codes N07BC01 or N07BC51) which, in Scandinavia, are almost solely prescribed for the treatment of opioid use disorder. Pre-pregnancy opioid use was defined as either having filled at least three prescriptions of opioid analgesics and 30 or more days of supply in the period of up to 12 months prior to pregnancy start (chronic opioid analgesics users) or having filled at least one prescription of any OMT drug in the period of up to 12 months prior to pregnancy or using illicit opioids before pregnancy (i.e., mothers filling prescriptions of OMT drugs during pregnancy, but not in the 12 months period before pregnancy) (Supplementary Figure S1). Information on illicit opioid use was not available, but since only mothers who had used illicit opioids before pregnancy would be initiated on OMT during pregnancy, we assumed that mothers receiving OMT during pregnancy were exposed to illicit opioids in the year before pregnancy. Pregnancy start was defined as the first day of the last menstrual period (LMP) and was ascertained using date of delivery and gestational age mainly based on prenatal ultrasound. Days’ supply was defined as the number of defined daily doses (DDDs) dispensed. The DDD is the assumed average maintenance dose per day for a drug used for its main indication in adults (WHO, 2021b).

### Comparison groups

For children born to mothers with chronic opioid analgesics use before pregnancy, we made three mutually exclusive comparison groups based on exposure to opioid analgesics during pregnancy, i.e., from pregnancy start until delivery (Supplementary Figure S1). Long-term maternal opioid analgesics use during pregnancy (i.e., *long-term exposed* children) was defined as being dispensed 30 or more days of supply during pregnancy, whereas short-term opioid analgesic use during pregnancy (i.e., *short-term exposed* children) was defined as being dispensed less than 30 days of supply. Opioid analgesics discontinuation (i.e., *unexposed* children) was defined as no filled prescriptions of opioids during pregnancy.

For children born to mothers with opioid use disorder (OMT cohort), we made two mutually exclusive comparison groups (Supplementary Figure S1). OMT exposed children were defined as children whose mothers filled at least one prescription of any OMT drug during pregnancy and who either filled OMT prescriptions before pregnancy or had illicit drug use before pregnancy. OMT unexposed children were defined as children whose mothers filled at least one prescription of any OMT drug in the 12 months prior to pregnancy and who did not fill any prescriptions of OMT drugs during pregnancy.

### Definition of outcome

Asthma was defined based on filled prescriptions of asthma drugs from the prescription registers and/or asthma diagnoses from the patient registers. In children 1 year of age or older, asthma was defined as two or more dispensed inhaled corticosteroids (ATC code R03BA), leukotriene receptor antagonists (R03DC), or fixed combinations of β2-agonists and corticosteroids (R03AK) within 365 days (and not on the same day) during follow-up time, or an asthma diagnosis (ICD-10 J45 or J46) as a primary or secondary diagnosis from specialist healthcare. The date of onset was set as the date when the child was defined as having asthma, i.e., the day of the first filled prescription within a 365-day period of any of the drugs or first date of diagnosis, whichever came first.

### Covariates

Information on maternal age, parity, smoking during pregnancy, cohabitation/marital status, sex of the child, year of birth and birth season was retrieved from the medical birth registers. Information on selected maternal diseases (asthma, reoccurring urinary tract infection, and hepatitis B and C) was retrieved from the medical birth registers, patient registers, and prescription registers (filled prescriptions of asthma drugs). Total number of any filled prescriptions, total number of pharmacological subgroups (ATC 3rd level) dispensed, total number of filled prescriptions for systemic treatment of infections (ATC group J), and dispensations (yes/no) of non-opioid analgesics (ATC groups N02B, N02C or M01), corticosteroids (ATC group H02A) and treatments for chronic obstructive pulmonary disease (ATC group R03) during pregnancy were retrieved from the prescription registers. All variables were assessed from pregnancy start until delivery, except for information on maternal chronic diseases from the patient registers that were assessed from 12 months before pregnancy start. Definitions of the covariates are shown in Supplementary Table S1.

### Missing data

We included only women with complete information on all covariates (complete case analysis).

### Statistical analysis

Characteristics of the mothers and newborns were summarized and described according to drug exposure. Crude cumulative incidence of asthma by age 4 years was calculated using the cumulative incidence function (CIF) to account for competing events. Cumulative incidence curves are presented in Supplementary Figure S2. The association between exposure and risk of childhood asthma was analyzed in children 1 year of age or older using Cox proportional hazard regression, with attained age as the time scale. We studied childhood asthma in children 1 year of age or older to avoid misclassification of the outcome in the youngest children as symptoms from respiratory infections can be misinterpreted as asthma, and also, antiasthmatic medication can be used to relieve such symptoms in young children. Individuals were censored at first study outcome, emigration (in Norway and Denmark), death, or end of study period, whichever occurred first. Hazard ratios (HRs) were calculated for long-term opioid analgesics exposed children versus short-term exposed, and long-term exposed versus unexposed, as well as OMT exposed children versus OMT unexposed. For the OMT comparison, restricted follow-up (730 days, i.e., 1–3 years of age) was applied for the pooled Norwegian/Swedish data due to few observations beyond 3 years of age (Supplementary Figures S3, S4). No restriction was applied for the OMT comparison for the Danish data.

Propensity scores were calculated to address imbalances in baseline confounder distributions. In logistic regression models, the probability of receiving treatment was estimated conditional on a set of identified confounders and risk factors for the outcome. A directed acyclic graph (DAG) showing the covariates included in the propensity score is shown in Supplementary Figure S5. We used the inverse probability of treatment weighting (IPTW) approach based on the propensity score to estimate hazard rate ratios associated with opioid use. Balance of baseline characteristics in the weighted population was assessed using the standardized mean difference. The standardized mean differences before and after applying the IPTW are included in the supplementary material (Supplementary Tables S3–S5). Normalized IPTW weights were included in adjusted Cox regression. Clustered robust variance estimators for 95% confidence intervals were used to account for clustering among mothers with multiple pregnancies.

For the opioid analgesics comparisons, adjusted HRs from Norway/Sweden and Denmark were pooled in a fixed-effects meta-analysis using the inverse variance method, that is weighting the country-specific log-hazard ratios (HRs) by the inverse of the within-countries’ variances. Meta-analysis was not performed for the OMT comparison since restricted follow-up was applied for the pooled Norwegian/Swedish data.

Statistical analyses were conducted using R (R Core Team, 2020). and SAS 9.4 (SAS Institute Inc., Cary, United States).

### Sensitivity analysis

Two sensitivity analyses were performed to evaluate potential misclassification of the outcome. In the first (sensitivity 1), the outcome was defined as two or more dispensed inhaled corticosteroids (R03BA), leukotriene receptor antagonists (R03DC), or fixed combinations of β2-agonists and corticosteroids (R03AK) within 365 days (and not on the same day) during follow-up time, and an asthma primary or secondary diagnosis (J45 or J46) (i.e., both filled prescriptions and diagnosis required). In the second (sensitivity 2), the outcome was defined as two or more dispensed inhaled corticosteroids (R03BA), leukotriene receptor antagonists (R03DC), or fixed combinations of β2-agonists and corticosteroids (R03AK) (not on the same day), or three or more dispensed selective β2-agonists (R03AC) within 365 days (on three different days) during follow-up time, or an asthma primary or secondary diagnosis (J45 or J46) (i.e., including short-acting β2-agonists). As an asthma diagnosis generally is more reliable, when set at a later age, we also performed a sensitivity analysis (sensitivity 3) where follow-up started at 6 years of age. To evaluate potential misclassification of OMT exposure we performed two additional analyses where OMT unexposed children with neonatal abstinence syndrome (NAS) were removed from the analysis (sensitivity 4) and where OMT unexposed children with NAS were defined as OMT exposed (sensitivity 5).

### Ethics

Use of data was approved by the Regional Ethical Research Board in Norway (2014/358/REK sør-øst D) and the Regional Ethical Review Board in Stockholm, Sweden (2009/775-31/4, 2016/152-32, and 2017/1159-32), and the Norwegian (16/01326-2/SBO) and the Danish Data Protection Agency (J.nr. 2013-41-1789). The national parliaments have on behalf of their populations given informed consent to be included in the registers. According to Danish legislation, no ethical permission is needed for registry-based research in Denmark.

## Results

### Baseline characteristics

Baseline characteristics of mothers and newborns are presented in Table 1. Among mothers who were chronic opioid analgesics users before pregnancy in the pooled Norwegian and Swedish cohort, those who were long-term exposed to opioids during pregnancy seemed to have a higher burden of health problems compared to those discontinuing use (unexposed) during pregnancy as shown by a higher number of pharmacological subgroups dispensed (64.2% vs. 32.9% were dispensed five or more subgroups) as well as more frequent use of other prescription drugs. In addition, long-term exposed mothers had higher parity (71.3% vs. 57.4% with 2 or more births) and higher prevalence of asthma (24.0% vs. 18.9%). Long-term opioid analgesics exposed mothers were more frequently smoking during pregnancy compared to those discontinuing use during pregnancy (30.2% vs. 21.2%). Mothers who were short-term exposed to opioids during pregnancy were found to be slightly healthier than the long-term exposed mothers. The observed patterns were similar in the Danish cohort. Neonatal abstinence syndrome (NAS) was more frequently reported for children long-term exposed to opioid analgesics (1.9% in Norway/Sweden and 8.2% in Denmark) compared to unexposed children (0.2% in Norway/Sweden and 0.8% in Denmark).

**TABLE 1:** Baseline characteristics of mothers and newborns stratified by comparison group.

	Norway/Sweden										Denmark									
	Opioid analgesics						OMT				Opioid analgesics						OMT			
	Long-term exposed		Short-term exposed		Unexposed		Exposed		Unexposed		Long-term exposed		Short-term exposed		Unexposed		Exposed		Unexposed	
	N	%	N	%	N	%	N	%	N	%	N	%	N	%	N	%	N	%	N	%
**Total**	3861	100	2027	100	3719	100	522	100	156	100	1229	100	979	100	2949	100	287	100	630	100
***Year of birth***																				
1997–2000	na	na	na	na	na	na	na	na	na	na	114	9,3	101	10,3	292	9,9	85	29,6	116	18,4
2001–2004	na	na	na	na	na	na	na	na	na	na	227	18,5	171	17,5	533	18,1	86	30,0	172	27,3
2005–2008	1236	32,0	558	27,5	1101	29,6	161	30,8	28	18,0	301	24,5	228	23,3	704	23,9	51	17,8	144	22,9
2009–2011	1461	37,8	718	35,4	1359	36,5	182	34,9	52	33,3	269	21,9	178	18,2	572	19,4	28	9,8	104	16,5
2012–2015	1164	30,2	751	37,1	1259	33,9	179	34,3	76	48,7	318	25,9	301	30,8	848	28,8	37	12,9	94	14,9
***Season of birth***																				
Spring/summer	1948	50,5	1036	51,1	1896	51,0	238	45,6	74	47,4	625	50,9	465	47,5	1325	44,9	132	46,0	322	51,1
Fall/winter	1913	49,6	991	48,9	1823	49,0	284	54,4	82	52,6	604	49,2	514	52,5	1624	55,1	155	54,0	308	48,9
***Sex of child***																				
Female	1880	48,7	1003	49,5	1865	50,2	261	50,0	75	48,1	612	49,8	476	48,6	1395	47,3	138	48,1	301	47,8
Male	1981	51,3	1024	50,5	1854	49,9	261	50,0	81	51,9	617	50,2	503	51,4	1554	52,7	149	51,9	329	52,2
***Maternal age***																				
< 35 years	2451	63,5	1433	70,7	2698	72,5	405	77,6	113	72,4	832	67,7	732	74,8	2194	74,4	222	77,4	453	71,9
≥ 35 years	1410	36,5	594	29,3	1021	27,5	117	22,4	43	27,6	397	32,3	247	25,2	755	25,6	65	22,7	177	28,1
***Parity***																				
1	1107	28,7	730	36,0	1584	42,6	228	43,7	57	36,5	399	32,5	392	40,0	1227	41,6	119	41,5	251	39,8
≥2	2754	71,3	1297	64,0	2135	57,4	294	56,3	99	63,5	813	66,2	569	58,1	1666	56,5	161	56,1	364	57,8
Missing	0	0,0	0	0,0	0	0,0	0	0,0	0	0,0	17	1,4	18	1,8	56	1,9	7	2,4	15	2,4
***Smoking in pregnancy***																				
No	2402	62,2	1283	63,3	2618	70,4	101	19,4	41	26,3	668	54,4	549	56,1	1941	65,8	32	11,2	118	18,7
Yes	1164	30,2	569	28,1	790	21,2	353	67,6	96	61,5	494	40,2	377	38,5	855	29,0	198	69,0	459	72,9
Missing	295	7,6	175	8,6	311	8,4	68	13,0	19	12,2	67	5,5	53	5,4	153	5,2	57	19,9	53	8,4
Civil status*																				
Not married	532	13,8	295	14,6	464	12,5	212	40,6	63	40,4	1055	85,8	839	85,7	2571	87,2	247	86,1	552	87,6
Married/co-habitant	3204	83,0	1691	83,4	3170	85,2	298	57,1	88	56,4	174	14,2	140	14,3	378	12,8	40	13,9	78	12,4
Missing	125	3,2	41	2,0	85	2,3	12	2,3	5	3,2	─	─	─	─	─	─	─	─	─	─
***Chronic maternal illness***																				
Asthma	926	24,0	424	20,9	704	18,9	102	19,5	29	18,6	213	17,3	154	15,7	407	13,8	44	15,3	87	13,8
Hepatitis	< 5	─	< 5	─	< 5	─	23	4,4	10	6,4	< 5	─	< 5	─	15	0,5	70	24,4	201	31,9
Reoccurring urinary tract infection	625	16,2	277	13,7	469	12,6	57	10,9	24	15,4	na	na	na	na	na	na	na	na	na	na
***Concomitant drug use during pregnancy***																				
Pharmacological subgroups**																				
0–1	116	3,0	96	4,7	719	19,3	161	30,8	63	40,4	260	21,2	213	21,8	1025	34,8	82	28,6	324	51,4
2	296	7,7	234	11,5	610	16,4	99	19,0	34	21,8	240	19,5	193	19,7	664	22,5	63	22,0	123	19,5
3	447	11,6	313	15,4	584	15,7	76	14,6	19	12,2	219	17,8	190	19,4	477	16,2	57	19,9	87	13,8
4	525	13,6	337	16,6	581	15,6	50	9,6	8	5,1	173	14,1	141	14,4	364	12,3	30	10,5	46	7,3
≥ 5	2477	64,2	1047	51,7	1225	32,9	136	26,1	32	20,5	337	27,4	242	24,7	419	14,2	55	19,2	50	7,9
No. of filled prescriptions																				
0–1	3	0,1	22	1,1	554	14,9	128	24,5	49	31,4	170	13,8	140	14,3	804	27,3	64	22,3	274	43,5
2–3	51	1,3	155	7,7	677	18,2	90	17,2	33	21,2	201	16,4	184	18,8	726	24,6	48	16,7	150	23,8
4–6	202	5,2	366	18,1	846	22,8	76	14,6	19	12,2	214	17,4	235	24,0	572	19,4	49	17,1	87	13,8
7–11	515	13,3	576	28,4	834	22,4	72	13,8	19	12,2	242	19,7	179	18,3	461	15,6	41	14,3	46	7,3
≥ 12	3090	80,0	908	44,8	808	21,7	156	29,9	36	23,1	402	32,7	241	24,6	386	13,1	85	29,6	73	11,6
No. of antiinfective prescriptions																				
0–1	2939	76,1	1555	76,7	3086	83,0	415	79,5	133	85,3	852	69,3	665	67,9	2174	73,7	226	78,8	501	79,5
2–3	642	16,6	363	17,9	495	13,3	81	15,5	18	11,5	265	21,6	229	23,4	567	19,2	50	17,4	116	18,4
≥ 4	280	7,3	109	5,4	138	3,7	26	5,0	5	3,2	112	9,1	85	8,7	208	7,1	11	3,8	13	2,1
Painkillers other than opiods	2234	57,9	825	40,7	789	21,2	65	12,5	16	10,3	625	50,9	404	41,3	628	21,3	51	17,8	59	9,4
Systemic corticosteriods	279	7,2	81	4,0	99	2,7	7	1,3	5	3,2	45	3,7	34	3,5	66	2,2	6	2,1	6	1,0
Drugs for treatment of COPD	717	18,6	270	13,3	419	11,3	77	14,8	14	9,0	151	12,3	104	10,6	271	9,2	48	16,7	60	9,5
***Neonatal characteristics***																				
NAS	74	1,9	9	0,4	8	0,2	280	53,6	34	21,8	101	8,2	12	1,2	23	0,8	183	63,8	100	15,9

OMT, opioid maintenance treatment; COPD, chronic obstructive pulmonary disease; NAS, neonatal abstinence syndrome.

* In Denmark, only information on marriage is available. Thus, those not registered as married are classified as not married. In Norway and Sweden, information on both marriage and cohabitation is available.**ATC 3rd level.

A large proportion of OMT exposed mothers and OMT discontinuers were smokers (OMT exposed: 67.6% in Norway/Sweden and 69.0% in Denmark). A higher proportion of the OMT exposed mothers were prescribed drugs belonging to five or more pharmacological subgroups and had a higher total number of any filled prescriptions during pregnancy compared with OMT discontinuers. The prevalence of hepatitis B and C virus infection was higher among OMT discontinuers (6.4% in Norway/Sweden and 31.9% in Denmark) compared to OMT exposed mothers. NAS was reported for more than half of the children in the OMT exposed cohort compared to 16% (Norway/Sweden) and 22% (Denmark) in the OMT unexposed cohort.

### Cumulative incidence of childhood asthma

Supplementary Figure S2 shows the unadjusted cumulative incidence curves for all exposure groups in Norway/Sweden and Denmark. Also, the 4-year cumulative incidence of asthma for all exposure groups is presented in Table 2.

**TABLE 2:** Cumulative incidence and propensity-score (PS) adjusted hazard ratios (HRs) for the association between prenatal opioid exposure and risk of childhood asthma.

	Norway and Sweden						Denmark						Meta-analysis
	Children (n)	Events (n)	Follow-up (person-years)	Cumulative incidence by age 4 yrs, % (95% CI)	Crude HR (95% CI)	Adjusted HR (95% CI)	Children (n)	Events (n)	Follow-up (person-years)	Cumulative incidence by age 4 yrs, % (95% CI)	Crude HR (95% CI)	Adjusted HR (95% CI)	Adjusted HR (95% CI)
***Opioid analgesics***													
Unexposed	2,979	561	10,349	18.5 (17.0–20.0)	ref	ref	2,744	581	1,7778	18.3 (16.9–19.8)	ref	ref	
Long-term exposed	3,492	786	11448	23.0 (21.5–24.5)	1.23 (1.10–1.38)	1.05 (0.88–1.26)	1,149	259	7,318	20.0 (17.7–22.2)	1.06 (0.91–1.24)	0.94 (0.79–1.11)	0.99 (0.87–1.12)
Short-term exposed	1,813	388	6,101	21.6 (19.5–23.6)	ref	ref	910	221	5,447	22.3 (19.7–25.0)	ref	ref	
Long-term exposed	3,492	787	11,444	23.0 (21.5–24.5)	1.06 (0.94–1.20)	1.05 (0.89–1.24)	1,149	259	7,318	20.0 (17.7–22.2)	0.89 (0.74–1.06)	0.86 (0.71–1.04)	0.96 (0.85–1.09)
OMT*													
OMT unexposed	1,34	21	193	17.7 (10.7–24.7)	ref	ref	563	108	4,747	14.5 (11.8–17.4)	ref	ref	
OMT exposed	443	73	696	20.0 (16.0–24.0)	0.98 (0.61–1.59)	1.13 (0.64–2.02)	226	58	1,902	20.0 (15.5–24.9)	1.39 (1.01–1.92)	1.25 (0.87–1.81)	na**

HR, hazard ratio; CI, confidence interval; OMT, opioid maintenance treatment.

*The OMT analyses were performed with the follow-up permitted by each data source. Follow-up was restricted to 730 days (i.e., age 1–3 years) for the pooled Norwegian/Swedish data since there were few observations beyond 730 days. For Denmark, no restriction was applied.**Meta-analysis was not performed due to restricted follow-up for the pooled Norwegian/Swedish data.

### Association of prenatal exposure to opioids and risk of childhood asthma

In the opioid analgesics cohort, the adjusted HR from the fixed-effects meta-analysis was 0.99 [95% CI 0.87-1.12] when long-term exposed children were compared with unexposed (Table 2). Similarly, when comparison was made between long- and short-term opioid analgesics exposed children, the adjusted HR was 0.96 (0.85–1.09). Details on country-specific estimates are presented in Table 2. In the Norwegian/Swedish OMT cohort the adjusted HR at 3 years of age was 1.13 (0.64–2.02) when OMT exposed children were compared with OMT unexposed children (Table 2). For the Danish OMT cohort, where no restriction of follow-up time was applied, the adjusted HR was 1.25 (0.87–1.81).

### Sensitivity analyses

**FIGURE 1:**
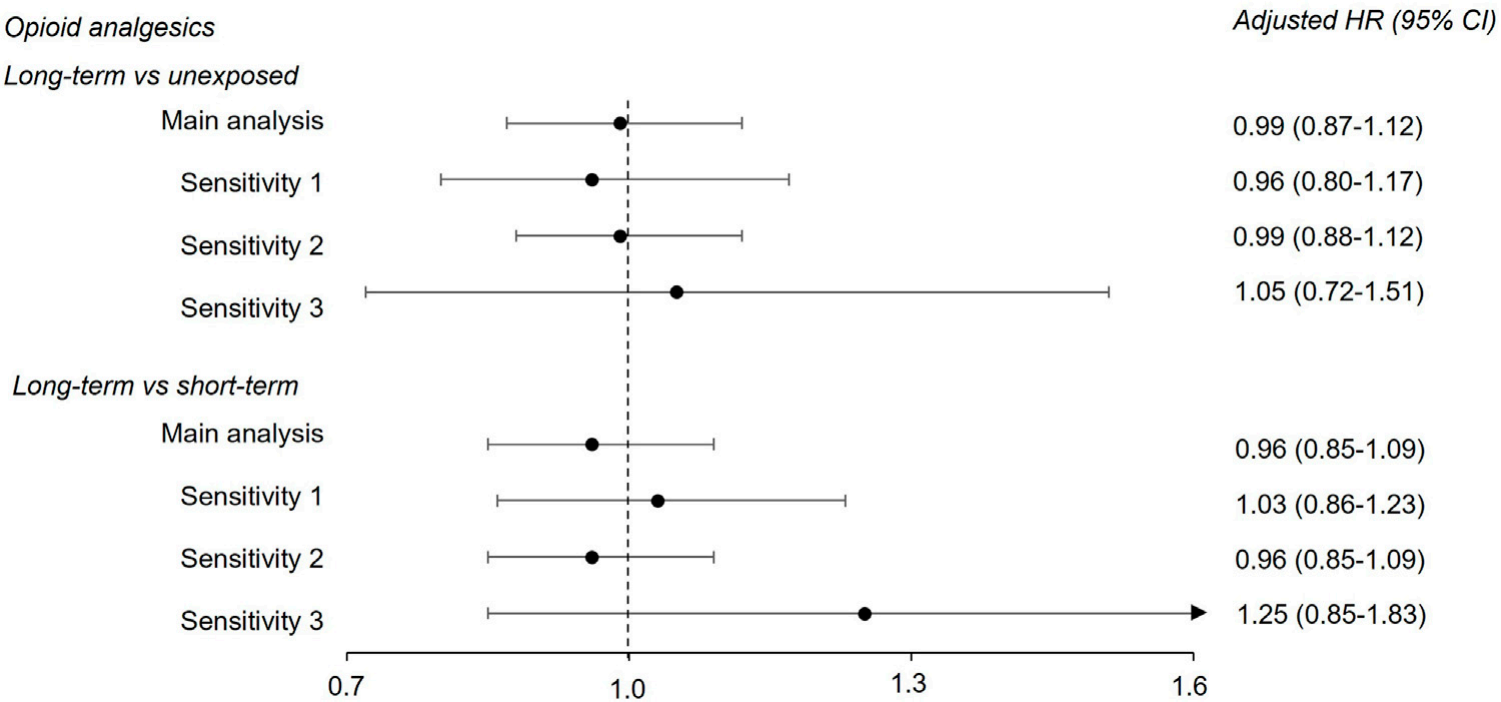
Adjusted hazard ratios (HR) and corresponding 95% confidence intervals (CI) from fixed-effects meta-analysis for the long-term opioid analgesics versus unexposed comparison and the long-term opioid analgesics versus short-term comparison. Results from main and sensitivity analyses are shown. See Table 2 and Supplementary Table S2 for country-specific estimates, as well as sensitivity analyses definitions.

**FIGURE 2:**
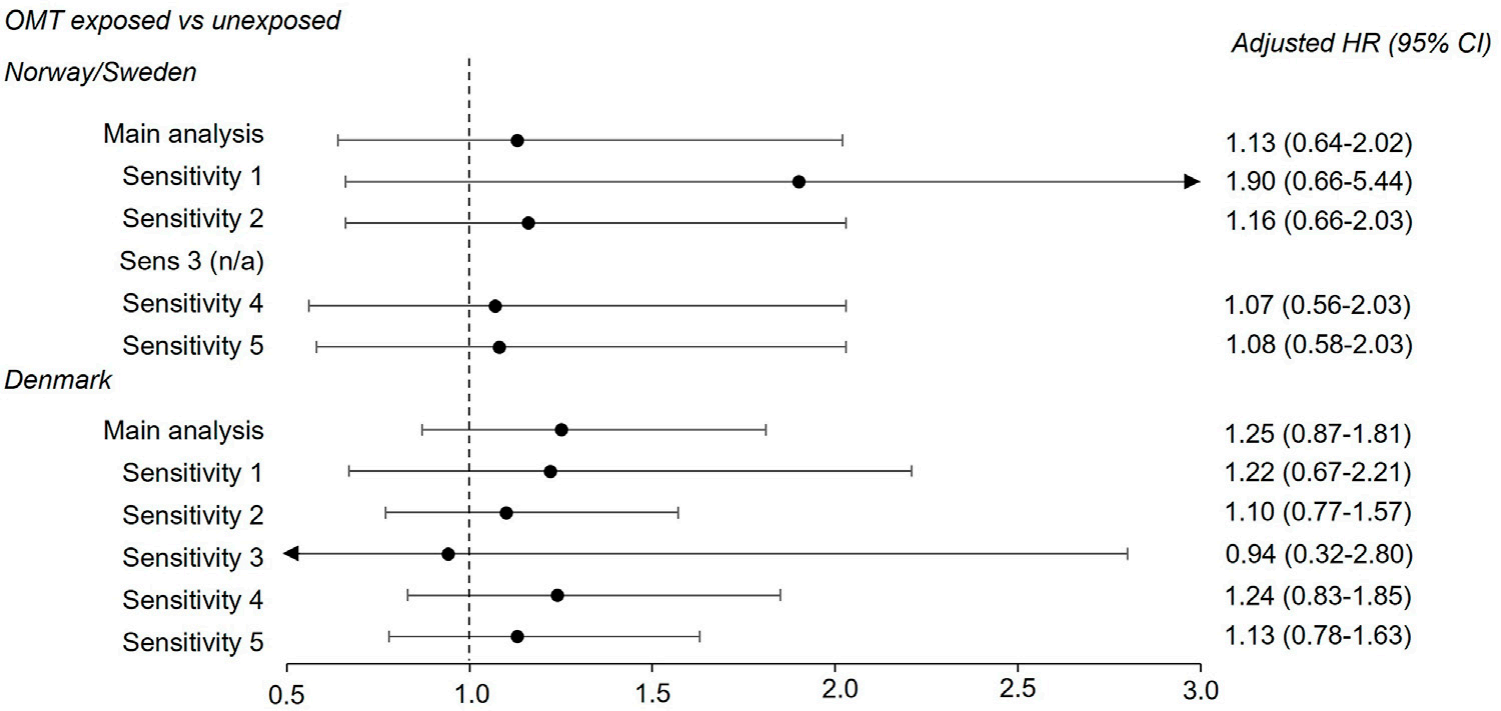
Adjusted hazard ratios (HR) and corresponding 95% confidence intervals (CI) from country-specific main and sensitivity analyses for the OMT exposed versus unexposed comparison. See footnote in Supplementary Table S2 for sensitivity analyses definitions. As follow-up was restricted at 3 years of age in the pooled data from Norway and Sweden, sensitivity analysis 3 (follow-up from 6 years of age) was not performed. OMT, opioid maintenance treatment.

For the opioid analgesic cohort, the estimates from the sensitivity analyses were in the same range as the main analysis (Figure 1). The point estimate was increased when follow-up started from 6 years of age (sensitivity 3), especially for the long-term exposed versus short-term exposed comparison. However, the estimate was imprecise [1.25 (0.85–1.83)]. For the OMT cohort the estimates were also in the same range as the main analysis, but with wider confidence intervals when we applied a stricter outcome definition (sensitivity 1), especially in the pooled data from Norway and Sweden [1.90 (0.66–5.44)] (Figure 2). When follow-up started from 6 years of age in the Danish data, the estimate also became less precise [0.94 (0.32–2.80)]. Details on country-specific estimates are presented in Supplementary Table S2.

## Discussion

In this Scandinavian cohort study with a total follow-up of up to 10 years in pooled data from Norway and Sweden and 19 years in Denmark, we report no significant increased risk of childhood asthma when comparing children born to mothers who were chronic opioid analgesic users before pregnancy and who either continued or discontinued use of opioids during pregnancy. No dose-response relationship was observed when comparing children long-term exposed to opioid analgesics with short-term exposed children. Results from the sensitivity analyses showed that the estimates of association were generally robust.

When comparing OMT exposed children with OMT unexposed, a non-significant increased risk of 25% was observed in Denmark. A non-significant increased risk of 13% was observed in children up to 3 years of age in the pooled data from Norway and Sweden. However, the upper bound of the confidence intervals ruled out more than a doubling of the risk. In the sensitivity analysis where we applied a stricter outcome definition, the estimates became imprecise especially in the pooled data from Norway and Sweden. When children with NAS, who were considered unexposed to OMT were reclassified as exposed, the estimates moved towards the null further supporting no association.

If there was a causal relationship between prenatal exposure to opioids and childhood asthma, we would have expected it to be reflected as a dose-response relationship when comparing children short-term or long-term exposed to opioid analgesics, unless there is a low threshold dose that triggers the effect. No such dose-response relationship was observed in our study. Also, no association was observed among children exposed to OMT, and thereby much higher opioid doses than for pain treatment, further supporting no dose-response relationship.

The Nordic countries have similar health systems and health- and population registers which makes it possible to perform large multi-database studies which is an advantage when studying rare exposures and rare outcomes (Selmer et al., 2016). Although the health systems and registers share many similarities, different treatment traditions and coding practices could lead to some variables being recorded differently or more accurately in one country than in the other countries. For the opioid analgesics cohorts, the prevalence of NAS was consistently higher in the Danish cohort. One possible explanation could be different opioid use patterns during pregnancy in the different countries. During the study period there were higher use of tramadol, oxycodone, and morphine among pregnant women in Denmark compared to Norway and Sweden where use of codeine-paracetamol was more common (Milada Mahic, personal communication). It has previously been shown that the risk of NAS is high for the opioids used in Denmark (Esposito et al., 2022). In our study we found that the prevalence of hepatitis was much higher in the OMT cohort in Denmark compared to the OMT cohort in Norway/Sweden. One possible explanation could be that the Danish cohort was comprised of women giving birth from the mid-1990s, while the Norwegian/Swedish cohort included births from mid-2000s. The introduction of direct-acting antivirals agents (DAAs) for treatment of hepatitis C infection were introduced from 2011 and has revolutionized treatment of hepatitis (Pecoraro et al., 2019). Thus, in the Danish cohort more women did not have access to such treatment.

Two previous population-based studies from Sweden have investigated the association between prenatal exposure to opioids and risk of childhood asthma. Källen *et al* found a positive association when adjusting for potential confounders, but the association was attenuated after restricting to women without asthma [OR = 1.56 (1.05-2.34)] showing that maternal asthma is an important confounder (Källén et al., 2013). Confounding by indication was not taken into consideration as children born to mothers exposed to opioids during pregnancy was compared with unexposed mothers with no requirement of pre-pregnancy opioid use. A recent population-based study from Sweden reported a positive association between prenatal opioid exposure and asthma/wheeze in children at the age of 4 years after adjusting for potential confounders [OR = 1.39 (1.30–1.49)]. However, when adjusting for intrinsic maternal factors, such as chronic pain and anxiety, in a sibling analysis, no association was observed [OR = 0.91 (0.62–1.31)] (Shaheen et al., 2019). The authors also investigated paracetamol and antimigraine drugs with similar results and concluded that the observed positive associations between prenatal exposure to analgesics and childhood asthma likely is confounded by maternal factors. Likewise, in a study from Norway an association between maternal pain in pregnancy and risk of childhood asthma was observed, further supporting maternal pain as an important confounder (Magnus et al., 2016).

To avoid confounding by indication it is important to choose relevant comparison groups. In our study we included women who were chronic opioid users before pregnancy, both women using opioid analgesics and women receiving OMT. The baseline characteristics of the various exposure groups indicated that the burden of health problems among these women correlated with the reason for use and treatment intensity. By applying propensity scores and a weighted approach, the observed differences in background characteristics were balanced and no associations were observed, further supporting the findings by Shaheen et al. (2019).

### Strengths and limitations

The study had several strengths. All live births in Denmark, Norway, and Sweden in the study period were included. Drug exposure was based on prospectively collected data on filled prescriptions from the nationwide prescription registers eliminating the possibility for recall bias and primary non-compliance (Furu et al., 2010). Confounding by indication was controlled for by only including women with chronic opioid analgesics use or use of OMT drugs or illicit opioid use before pregnancy. For the opioid analgesics comparisons, it was important to exclude women using opioids to treat acute pain since they might have a different health status than women using opioid analgesics long-term. Opioid use with two different indications were explored, namely, pain and opioid use disorder, representing women with different confounder distributions and different treatment intensity. Women receiving OMT were studied separately since opioid use disorder is associated with worse health outcomes and more unstable living conditions that could potentially lead to bias of the result if this population was compared with women not receiving OMT. By using propensity scores, the comparison groups were balanced across several potential important confounders making it possible to come closer to unbiased associations.

The study had some limitations. Since exposure is based on filled prescriptions, we do not know whether the women used the drugs. However, by only including chronic opioid analgesics users, the women had filled several prescriptions or been dispensed a large amount of opioids before pregnancy, increasing the probability that they were consuming the drugs. Timing of exposure in pregnancy, which may be important particularly for exposure during early development when the immune system is developing, could not be assessed due to small sample size. We also did not assess exposure to individual opioids so any drug specific effects could be masked. Further, regarding OMT exposure, some women might receive OMT drugs from specialized addiction outpatient clinics within specialist care. As the prescription registers only contain information about filled prescriptions from pharmacies this might lead to selection bias since some OMT exposed children might not be captured. It could also lead to misclassification of exposure as some women retrieving OMT drugs from pharmacies before pregnancy might have a closer follow-up in specialized outpatient clinics during pregnancy and their OMT drug use during pregnancy would not be captured. We tried to overcome this limitation by performing sensitivity analyses to address potential misclassification of OMT exposure. We did not have information about illicit opioid use which could be an issue particularly for the OMT analyses.

Identifying asthma in children is challenging since, particularly in small children, symptoms of respiratory infections could be misinterpreted as asthma and antiasthmatic medications could be used to relieve such symptoms. We performed a range of sensitivity analyses to address potential misclassification of the outcome.

Even though we tried to identify women with chronic pain via chronic opioid analgesics use, we did not have information about the severity of pain that could have an impact on the observed associations. Also, we had no information about whether the women had planned to become pregnant, but it is estimated that 38% of pregnancies in Northern Europe are unintended (Sedgh et al., 2014). A planned pregnancy might indicate a healthier lifestyle, and this could also be the reason for discontinuing drug use. We tried to overcome this by using propensity scores to balance the measured covariates, but there could still be differences in unmeasured covariates.

## Conclusion

We found no association between prenatal exposure to opioids and risk of childhood asthma. Also, we did not observe any dose-response relationship for opioid exposure. The results were consistent across two different opioid exposure groups with different confounder distributions. Our findings are reassuring for women who need to use opioids in pregnancy. The study shows the importance of choosing relevant comparison groups and applying appropriate statistical methods to control for confounding by indication.

## Data Availability

The data analyzed in this study is subject to the following licenses/restrictions: The register-based cohorts are based on individual-level data from national health and population registers. The authors are not allowed, by law, to publicly share this data. Therefore, the authors cannot make this data fully available to the public. The authors may share statistical code. Requests to access these datasets should be directed to the register holders in each country.
