# Supplementary material for: Prenatal opioid exposure and risk of asthma in childhood: a population-based study from Denmark, Norway, and Sweden
Source: Front Pharmacol. 2023 May 4;14:1056192. doi: 10.3389/fphar.2023.1056192 (PMC10192698; doi:10.3389/fphar.2023.1056192)
Supplement: Supplementary file 1 [file DataSheet2.docx]

**Supplementary Table S1.** Covariate definitions

| **Covariate** | **Definition** |
| --- | --- |
| Year of birth | As recorded in MBR |
| Season of birth | As recorded in MBR |
| Sex of child | As recorded in MBR |
| Maternal age at delivery | As recorded in MBR |
| Parity | As recorded in MBR |
| Smoking in pregnancy | As recorded in MBR |
| Civil status | As recorded in MBR  (In Denmark, only information on marriage is available. Thus, those not registered as married are classified as not married. In Norway and Sweden, information on both marriage and cohabitation is available). |
| Maternal asthma | Recorded in MBR or NPR (ICD-10): J45, J46  Recorded in KUHR (Norway) (ICPC-2): R96  Recorded in PDR (ATC): at least 2 dispensations of R03AK, R03BA, or R03DC within 365 days |
| Hepatitis | Recorded in MBR or NPR (ICD-10): B16-B19 |
| Reoccuring urinary tract infection | Recorded in MBR (not available in Denmark) |
| Number of pharmacological subgroups | Recorded in PDR: ATC 3rd level |
| Number of filled prescriptions | Recorded in PDR |
| Number of filled prescriptions for systemic treatment of infections | Recorded in PDR (ATC): J |
| Painkillers other than opioids | Recorded in PDR (ATC): N02B, N02C, or M01 |
| Systemic corticosteriods | Recorded in PDR (ATC): H02A |
| Treatments for chronic obstructive pulmonary disease | Recorded in PDR (ATC): R03 |
| NAS | As recorded in MBR |
| MBR=medical birth register; NPR=national patient register; KUHR=the Control and Payment of Health Reimbursement Database; PDR=prescribed drug register; ICD-10=International Classification of Diseases, 10th revision; ICPC-2=International Classification of Primary Care, 2nd edition; ATC=anatomic therapeutic chemical code; NAS= neonatal abstinence syndrome | |

**Supplementary Table S2** Crude and propensity-score (PS) adjusted hazard ratios (HRs) for the association between prenatal opioid exposure and risk of childhood asthma stratified by type of sensitivity analysis.

|  |  | Norway and Sweden | | | | |  | Denmark | | | | |  | Meta-analysis |
| --- | --- | --- | --- | --- | --- | --- | --- | --- | --- | --- | --- | --- | --- | --- |
|  |  | Children (n) | Events (n) | Follow-up (person-years) | Crude HR (95% CI) | Adjusted HR (95% CI) |  | Children (n) | Events (n) | Follow-up (person-years) | Crude HR (95% CI) | Adjusted HR (95% CI) |  | Adjusted HR (95% CI) |
|  | ***Opioid analgesics*** |  |  |  |  |  |  |  |  |  |  |  |  |  |
| Sensitivity 1 | Unexposed | 2979 | 298 | 11158 | ref | ref |  | 2744 | 175 | 20395 | ref | ref |  |  |
|  | Long-term exposed | 3492 | 449 | 12481 | 1.31 (1.13-1.52) | 1.03 (0.81-1.31) |  | 1149 | 78 | 8580 | 1.05 (0.80-1.39) | 0.86 (0.63-1.18) |  | 0.96 (0.80-1.17) |
| Sensitivity 2 | Unexposed | 2979 | 579 | 10265 | ref | ref |  | 2744 | 608 | 17625 | ref | ref |  |  |
|  | Long-term exposed | 3492 | 798 | 11403 | 1.21 (1.08-1.35) | 1.04 (0.87-1.25) |  | 1149 | 275 | 7193 | 1.08 (0.93-1.25) | 0.96 (0.81-1.13) |  | 0.99 (0.88-1.12) |
| Sensitivity 3 | Unexposed | 1137 | 80 | 1920 | ref | ref |  | 1319 | 43 | 6981 | ref | ref |  |  |
|  | Long-term exposed | 1333 | 116 | 2037 | 1.29 (0.96-1.72) | 1.07 (0.67-1.71) |  | 543 | 19 | 2708 | 1.14 (0.67-1.97) | 1.01 (0.56-1.83) |  | 1.05 (0.72-1.51) |
|  |  |  |  |  |  |  |  |  |  |  |  |  |  |  |
| Sensitivity 1 | Short-term exposed | 1813 | 208 | 6670 | ref | ref |  | 910 | 73 | 6523 | ref | ref |  |  |
|  | Long-term exposed | 3492 | 450 | 12477 | 1.13 (0.96-1.34) | 1.15 (0.92-1.43) |  | 1149 | 78 | 8580 | 0.82 (0.59-1.14) | 0.79 (0.56-1.10) |  | 1.03 (0.86-1.23) |
| Sensitivity 2 | Short-term exposed | 1813 | 399 | 6064 | ref | ref |  | 910 | 236 | 5354 | ref | ref |  |  |
|  | Long-term exposed | 3492 | 799 | 11399 | 1.04 (0.92-1.18) | 1.05 (0.89-1.23) |  | 1149 | 275 | 7193 | 0.88 (0.74-1.05) | 0.85 (0.71-1.03) |  | 0.96 (0.85-1.09) |
| Sensitivity 3 | Short-term exposed | 684 | 60 | 1184 | ref | ref |  | 394 | 8 | 2038 | ref | ref |  |  |
|  | Long-term exposed | 1333 | 116 | 2037 | 1.04 (0.76-1.43) | 1.17 (0.76-1.78) |  | 543 | 19 | 2708 | 1.79 (0.78-4.09) | 1.69 (0.68-4.17) |  | 1.25 (0.85-1.83) |
|  |  |  |  |  |  |  |  |  |  |  |  |  |  |  |
|  | ***OMT**** |  |  |  |  |  |  |  |  |  |  |  |  |  |
| Sensitivity 1 | Unexposed | 134 | 6 | 209 | ref | ref |  | 563 | 33 | 5298 | ref | ref |  |  |
|  | Exposed | 443 | 39 | 733 | 1.90 (0.80-4.54) | 1.90 (0.66-5.44) |  | 226 | 18 | 2248 | 1.35 (0.78-2.34) | 1.22 (0.67-2.21) |  | n/a** |
| Sensitivity 2 | Unexposed | 134 | 22 | 192 | ref | ref |  | 563 | 120 | 4661 | ref | ref |  |  |
|  | Exposed | 443 | 77 | 691 | 0.99 (0.62-1.59) | 1.16 (0.66-2.03) |  | 226 | 60 | 1882 | 1.28 (0.94-1.75) | 1.10 (0.77-1.57) |  | n/a** |
| Sensitivity 3 | Unexposed | n/a | n/a | n/a | n/a | n/a |  | 358 | 17 | 2182 | ref | ref |  |  |
|  | Exposed | n/a | n/a | n/a | n/a | n/a |  | 130 | 6 | 920 | 0.84 (0.34-2.08) | 0.94 (0.32-2.80) |  | n/a** |
| Sensitivity 4 | Unexposed | 105 | 16 | 152 | ref | ref |  | 482 | 94 | 4018 | ref | ref |  |  |
|  | Exposed | 443 | 73 | 696 | 1.01 (0.59-1.74) | 1.07 (0.56-2.03) |  | 226 | 58 | 1902 | 1.36 (0.98-1.89) | 1.24 (0.83-1.85) |  | n/a** |
| Sensitivity 5 | Unexposed | 105 | 16 | 152 | ref | ref |  | 482 | 94 | 4018 | ref | ref |  |  |
|  | Exposed | 472 | 78 | 737 | 1.02 (0.60-1.75) | 1.08 (0.58-2.03) |  | 307 | 72 | 2630 | 1.23 (0.90-1.67) | 1.13 (0.78-1.63) |  | n/a** |

n=number of children; HR=hazard ratio; CI=confidence interval; OMT=opioid maintenance treatment

Sensitivity 1: Outcome = 2 x (R03BA | R03DC | R03AK) on 2 different days within 365 days AND 1 x (J45 | J46) in patient register, follow-up from age 1

Sensitivity 2: Outcome = 2 x (R03BA | R03DC | R03AK) on 2 different days within 365 days OR 1 x (J45 | J46) in patient register OR 3 x R03AC on 3 different days within 365 days, follow-up from age 1

Sensitivity 3: Outcome = 2 x (R03BA | R03DC | R03AK) on 2 different days within 365 days OR 1 x (J45 | J46) in patient register, follow-up from age 6

Sensitivity 4: OMT unexposed children with NAS removed

Sensitivity 5: OMT unexposed children with NAS defined as OMT exposed

*The OMT analyses were performed with the follow-up permitted by each data source. Follow-up was restricted to 730 days (i.e. age 1-3 years) for the pooled Norwegian/Swedish data since there were few observations beyond 730 days. For Denmark, no restriction was applied.

**Meta-analysis was not performed due to restricted follow-up for the pooled Norwegian/Swedish data.

| **Supplementary Table S3** Co-variate specific standardized mean difference (SMD) before and after propensity-score adjustment. Comparison long-term opioid analgesics exposed vs unexposed. | | | | | | | |
| --- | --- | --- | --- | --- | --- | --- | --- |
|  |  |  |  |  |  |  |  |
|  | Norway and Sweden | |  | Denmark | |  |  |
| Characteristic | SMD before adjustment | SMD after adjustment |  | SMD before adjustment | SMD after adjustment |  |  |
| *Year of birth* |  |  |  |  |  |  |  |
| 1997-2000 | na | na |  | -0.026 | -0.013 |  |  |
| 2001-2004 | na | na |  | 0.006 | -0.020 |  |  |
| 2005-2008 | na | na |  | 0.020 | -0.016 |  |  |
| 2009-2011 | na | na |  | 0.062 | 0.042 |  |  |
| 2012-2015 | na | na |  | -0.065 | 0.002 |  |  |
| Born after 2011 | -0.04 | 0.019 |  | na | na |  |  |
| Born in Norway | -0.155 | -0.013 |  | na | na |  |  |
| Born in spring | -0.001 | 0.005 |  | 0.115 | 0.001 |  |  |
| Male sex | 0.017 | -0.01 |  | 0.045 | 0.007 |  |  |
| Maternal age (≥35 years) | 0.051 | 0.008 |  | -0.137 | -0.004 |  |  |
| Primiparous | -0.134 | -0.012 |  | -0.191 | 0.006 |  |  |
| Smoking in pregnancy (yes) | 0.095 | 0.018 |  | 0.247 | -0.014 |  |  |
| Cohabitant/ married | -0.013 | 0.008 |  | 0.042 | -0.029 |  |  |
| Maternal asthma | 0.049 | -0.005 |  | 0.098 | 0.001 |  |  |
| Hepatitis | 0 | 0 |  | -0.073 | 0.000 |  |  |
| Reoccurring urinary tract infection | 0.039 | 0.004 |  | na | na |  |  |
| *Number of pharmacological subgroups* | |  |  |  |  |  |  |
| 0-1 | 0.165 | 0.016 |  | -0.297 | -0.020 |  |  |
| 2 | -0.084 | -0.026 |  | -0.082 | -0.001 |  |  |
| 3 | -0.041 | 0.023 |  | 0.045 | -0.006 |  |  |
| 4 | -0.023 | -0.015 |  | 0.043 | 0.015 |  |  |
| ≥ 5 | 0.311 | 0.034 |  | 0.335 | 0.013 |  |  |
| *Total number of prescriptions* |  |  |  |  |  |  |  |
| 0-1 | 0.147 | 0.009 |  | -0.329 | 0.000 |  |  |
| 2-3 | -0.168 | -0.032 |  | -0.208 | -0.013 |  |  |
| 4-6 | -0.173 | -0.009 |  | -0.039 | -0.006 |  |  |
| 7-11 | -0.095 | 0.022 |  | 0.118 | -0.012 |  |  |
| ≥ 12 | 0.583 | 0.028 |  | 0.458 | 0.025 |  |  |
| *Number of antiinfective prescriptions* |  |  |  |  |  |  |  |
| 0-1 | 0.067 | 0.023 |  | -0.101 | 0.007 |  |  |
| 2-3 | 0.033 | 0.015 |  | 0.068 | -0.019 |  |  |
| ≥ 4 | 0.035 | 0.009 |  | 0.067 | 0.016 |  |  |
| Painkillers other than opiods | 0.366 | 0.058 |  | 0.631 | 0.010 |  |  |
| Systemic corticosteriods | 0.046 | -0.002 |  | 0.071 | -0.009 |  |  |
| Drugs for treatment of COPD | 0.072 | 0.004 |  | 0.100 | 0.014 |  |  |
| OMT=opioid maintenance treatment; COPD=chronic obstructive pulmonary disease | | | | | |  |  |

| **Supplementary Table S4** Co-variate specific standardized mean difference (SMD) before and after propensity-score adjustment. Comparison long-term vs short-term opioid analgesics exposed. | | | | | | | |
| --- | --- | --- | --- | --- | --- | --- | --- |
|  |  |  |  |  |  |  |  |
|  | Norway and Sweden | |  | Denmark | |  |  |
| Characteristic | SMD before adjustment | SMD after adjustment |  | SMD before adjustment | SMD after adjustment |  |  |
| *Year of birth* |  |  |  |  |  |  |  |
| 1997-2000 | na | na |  | 0.033 | -0.002 |  |  |
| 2001-2004 | na | na |  | -0.034 | -0.002 |  |  |
| 2005-2008 | na | na |  | -0.034 | 0.008 |  |  |
| 2009-2011 | na | na |  | -0.074 | -0.003 |  |  |
| 2012-2015 | na | na |  | 0.107 | -0.002 |  |  |
| Born after 2011 | -0.070 | 0.007 |  | na | na |  |  |
| Born in Norway | -0.225 | -0.007 |  | na | na |  |  |
| Born in spring | -0.018 | -0.015 |  | -0.078 | 0.005 |  |  |
| Male sex | -0.003 | 0 |  | -0.022 | 0.013 |  |  |
| Maternal age (≥ 35 years) | 0.019 | -0.005 |  | 0.145 | -0.009 |  |  |
| Primiparous | -0.071 | -0.003 |  | 0.147 | 0.019 |  |  |
| Smoking in pregnancy (yes) | 0.019 | 0.003 |  | -0.042 | -0.008 |  |  |
| Cohabitant/ married | 0.006 | -0.01 |  | 0.001 | -0.013 |  |  |
| Maternal asthma | 0.030 | 0 |  | -0.033 | 0.001 |  |  |
| Hepatitis | -0.001 | 0 |  | 0.053 | 0.009 |  |  |
| Reoccurring urinary tract infection | 0.029 | 0.015 |  | na | na |  |  |
| *Number of pharmacological subgroups* |  |  |  |  |  |  |  |
| 0-1 | 0.018 | -0.008 |  | 0.000 | -0.002 |  |  |
| 2 | -0.040 | 0.008 |  | 0.008 | 0.011 |  |  |
| 3 | -0.038 | 0.017 |  | 0.026 | -0.008 |  |  |
| 4 | -0.032 | -0.021 |  | 0.030 | -0.016 |  |  |
| ≥ 5 | 0.128 | -0.011 |  | -0.054 | 0.013 |  |  |
| *Total number of prescriptions* |  |  |  |  |  |  |  |
| 0-1 | 0.009 | 0.001 |  | -0.005 | 0.000 |  |  |
| 2-3 | -0.066 | -0.01 |  | 0.057 | 0.008 |  |  |
| 4-6 | -0.129 | -0.011 |  | 0.159 | -0.013 |  |  |
| 7-11 | -0.152 | 0.001 |  | -0.032 | -0.006 |  |  |
| ≥ 12 | 0.357 | 0.022 |  | -0.161 | 0.012 |  |  |
| *Number of antiinfective prescriptions* |  |  |  |  |  |  |  |
| 0-1 | 0.004 | 0.006 |  | -0.030 | 0.017 |  |  |
| 2-3 | -0.012 | 0.005 |  | 0.038 | -0.014 |  |  |
| ≥ 4 | 0.017 | 0 |  | -0.007 | -0.008 |  |  |
| Painkillers other than opiods | 0.174 | 0.011 |  | -0.178 | 0.003 |  |  |
| Systemic corticosteriods | 0.032 | -0.012 |  | 0.007 | -0.001 |  |  |
| Drugs for treatment of COPD | 0.055 | -0.002 |  | -0.045 | 0.011 |  |  |
| OMT=opioid maintenance treatment; COPD=chronic obstructive pulmonary disease | | | | | |  |  |

| **Supplementary Table S5** Co-variate specific standardized mean difference (SMD) before and after propensity-score adjustment. Comparison OMT exposed vs OMT unexposed. | | | | | | | | |
| --- | --- | --- | --- | --- | --- | --- | --- | --- |
|  |  |  |  |  |  |  |  |  |
|  | Norway and Sweden | |  | Denmark | |  |  |  |
| Characteristic | SMD before adjustment | SMD after adjustment |  | SMD before adjustment | SMD after adjustment |  |  |  |
| *Year of birth* |  |  |  |  |  |  |  |  |
| 1997-2000 | na | na |  | 0.033 | -0.002 |  |  |  |
| 2001-2004 | na | na |  | -0.034 | -0.002 |  |  |  |
| 2005-2008 | na | na |  | -0.034 | 0.008 |  |  |  |
| 2009-2011 | na | na |  | -0.074 | -0.003 |  |  |  |
| 2012-2015 | na | na |  | 0.107 | -0.002 |  |  |  |
| Born after 2011 | -0.159 | 0.010 |  | na | na |  |  |  |
| Born in Norway | 0.057 | 0.011 |  | na | na |  |  |  |
| Born in spring | -0.011 | -0.042 |  | -0.078 | 0.005 |  |  |  |
| Male sex | -0.016 | -0.004 |  | -0.022 | 0.013 |  |  |  |
| Maternal age (≥ 35 years) | -0.061 | -0.010 |  | 0.145 | -0.009 |  |  |  |
| Primiparous | 0.068 | 0.006 |  | 0.147 | 0.019 |  |  |  |
| Smoking in pregnancy (yes) | 0.075 | 0.017 |  | -0.042 | -0.008 |  |  |  |
| Cohabitant/ married | -0.007 | -0.037 |  | 0.001 | -0.013 |  |  |  |
| Maternal asthma | 0.002 | 0.004 |  | -0.033 | 0.001 |  |  |  |
| Hepatitis | -0.024 | 0.006 |  | 0.053 | 0.009 |  |  |  |
| Reoccurring urinary tract infection | -0.053 | -0.013 |  | na | na |  |  |  |
| *Number of pharmacological subgroups* |  |  |  |  |  |  |  |  |
| 0-1 | 0.101 | -0.011 |  | 0.000 | -0.002 |  |  |  |
| 2 | -0.012 | -0.010 |  | 0.008 | 0.011 |  |  |  |
| 3 | 0.041 | -0.013 |  | 0.026 | -0.008 |  |  |  |
| 4 | 0.049 | 0.013 |  | 0.030 | -0.016 |  |  |  |
| ≥ 5 | 0.022 | 0.000 |  | -0.054 | 0.013 |  |  |  |
| *Total number of prescriptions* |  |  |  |  |  |  |  |  |
| 0-1 | 0.073 | -0.021 |  | -0.005 | 0.000 |  |  |  |
| 2-3 | -0.037 | -0.026 |  | 0.057 | 0.008 |  |  |  |
| 4-6 | 0.038 | 0.001 |  | 0.159 | -0.013 |  |  |  |
| 7-11 | 0.022 | -0.008 |  | -0.032 | -0.006 |  |  |  |
| ≥ 12 | 0.050 | 0.013 |  | -0.161 | 0.012 |  |  |  |
| *Number of antiinfective prescriptions* |  |  |  |  |  |  |  |  |
| 0-1 | 0.052 | -0.021 |  | -0.030 | 0.017 |  |  |  |
| 2-3 | 0.037 | -0.009 |  | 0.038 | -0.014 |  |  |  |
| ≥ 4 | 0.015 | -0.012 |  | -0.007 | -0.008 |  |  |  |
| Painkillers other than opiods | 0.024 | 0.006 |  | -0.178 | 0.003 |  |  |  |
| Systemic corticosteriods | -0.014 | 0.000 |  | 0.007 | -0.001 |  |  |  |
| Drugs for treatment of COPD | 0.049 | 0.010 |  | -0.045 | 0.011 |  |  |  |
| OMT=opioid maintenance treatment; COPD=chronic obstructive pulmonary disease | | | | | |  |  |  |
