# Supplementary material for: Prenatal opioid exposure and risk of asthma in childhood: a population-based study from Denmark, Norway, and Sweden
Source: Front Pharmacol. 2023 May 4;14:1056192. doi: 10.3389/fphar.2023.1056192 (PMC10192698; doi:10.3389/fphar.2023.1056192)
Supplement: Supplementary file 2 [file DataSheet1.docx]

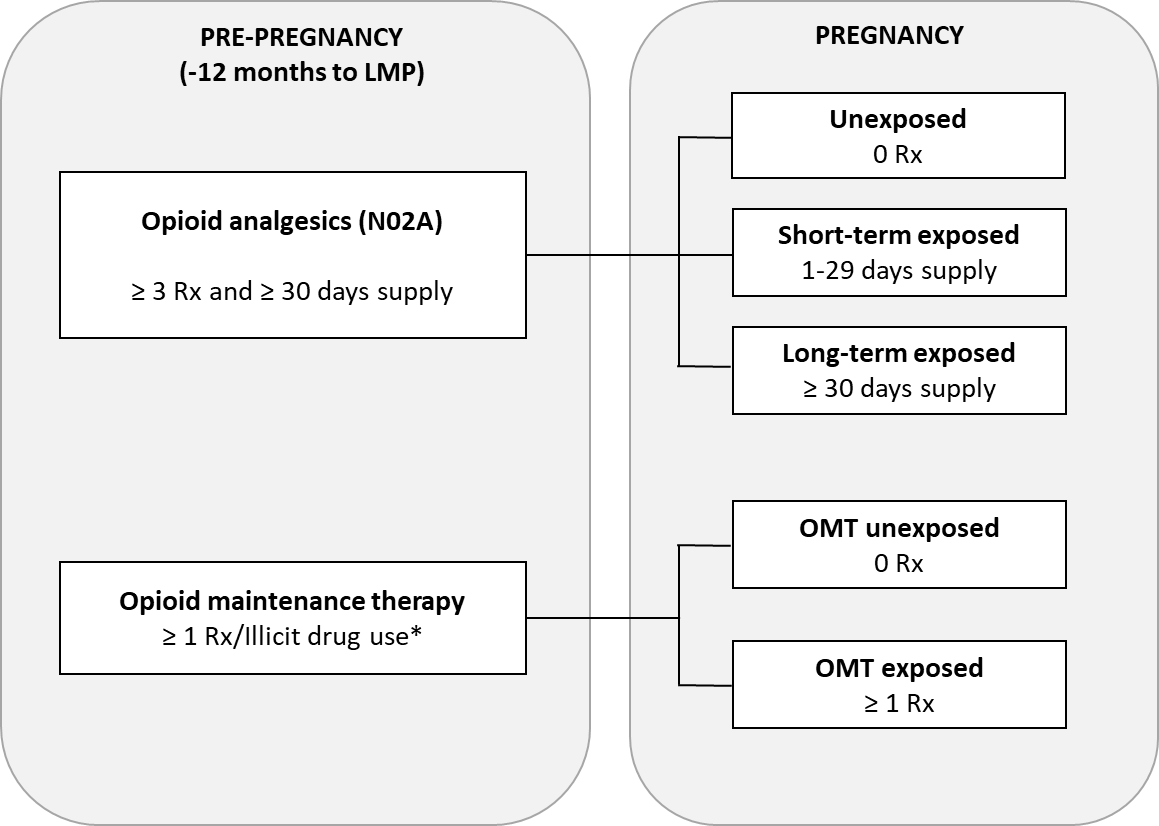


**Supplementary Figure S1.** Schematic of pre-pregnancy opioid use and mutually exclusive comparison groups.

For pre-pregnancy chronic opioid analgesics users, three mutually exclusive comparison groups were made; unexposed, short-term exposed and long-term exposed during pregnancy. For women receiving OMT, two mutually exclusive comparison groups were made; OMT unexposed and OMT exposed during pregnancy.

* To be eligible for inclusion in the OMT unexposed group, the mother had to have at least one filled OMT prescription in the 12 months before pregnancy. For the OMT exposed group, the mother could either have filled prescriptions before pregnancy or have illicit opioid use, i.e., mothers filling prescriptions of OMT drugs during pregnancy, but not in the 12 months period before pregnancy. Information on illicit opioid use was not available, but since only mothers who had used illicit opioids before pregnancy would be initiated on OMT during pregnancy, we assumed that they were exposed to illicit opioids in the year before pregnancy.

LMP = last menstrual period; Rx = dispensed prescription; OMT = opioid maintenance treatment.

**Supplementary Figure S2.** Unadjusted cumulative incidence curves for the pooled Norwegian/Swedish data (A) and the Danish data (B).

**A) Norway/Sweden**


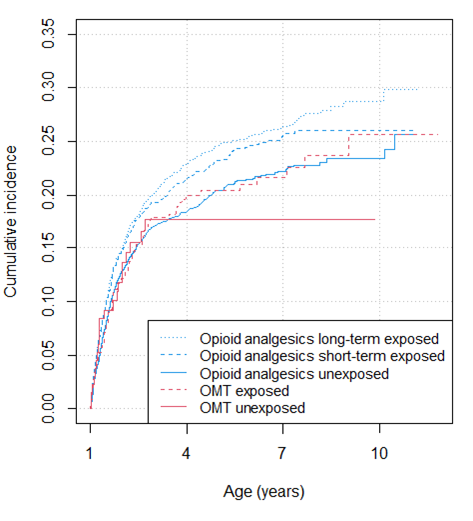


**B) Denmark**


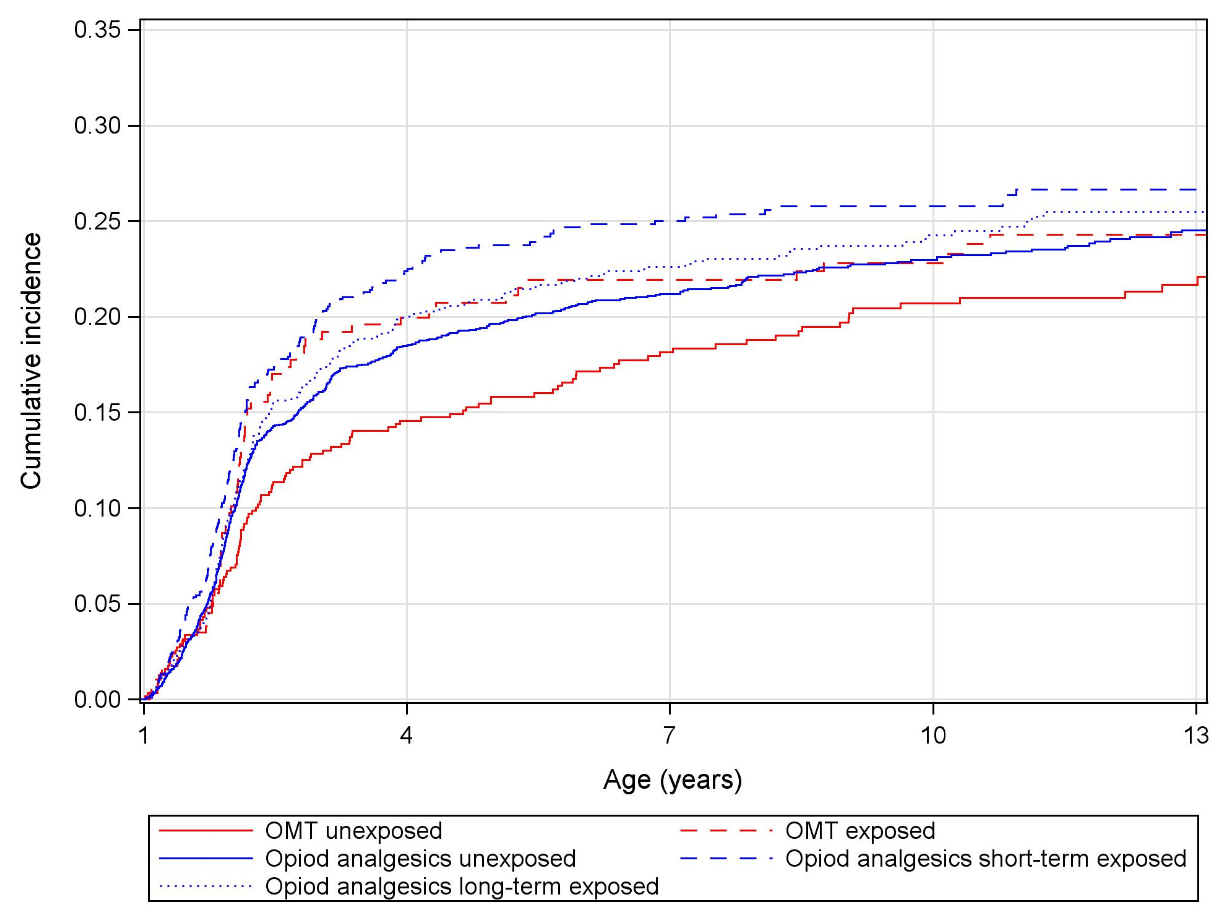


**Supplementary Figure S3.** Unadjusted Kaplan-Meier curves for the pooled Norwegian/Swedish data (time 0 = 365 days, i.e., 1 year of age; unit on x-axis = days). Number of children at risk is shown below the graph. p-values from the proportional hazard assumption test for exposure are shown. The p-values were tested with significant level alpha=0.05. The proportionality assumption does not fail if the p-values are greater than the significant level.

**A) Long-term opioid analgesics exposed vs unexposed**


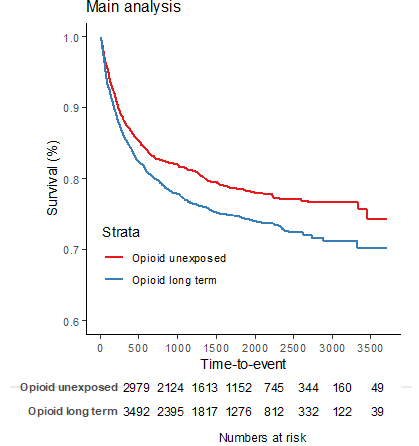


**B) Long-term opioid analgesics exposed vs short-term exposed**


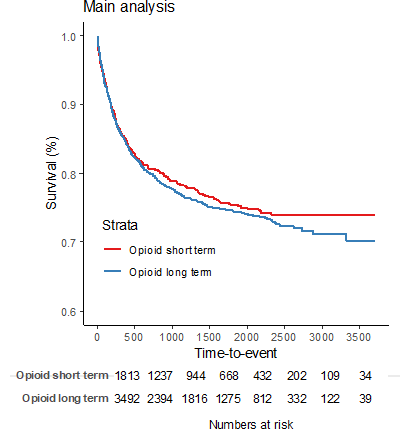


**C) OMT exposed vs OMT unexposed**


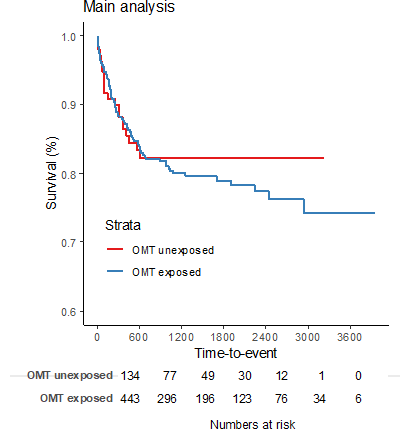


The PH assumption did not hold for the OMT comparison for the pooled data from Norway and Sweden due to no events beyond 730 days follow-up (time 0 = 365 days, i.e., 1 year of age). This could be explained by few children at risk. We decided to restrict the follow-time to 730 days (i.e., children 1-3 years of age) for the OMT analysis for the pooled Norwegian/Swedish data:


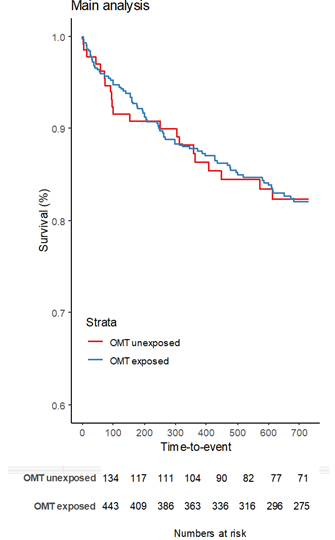


The PH assumption was not violated for the OMT analysis with restricted follow-up of 730 days.

**Supplementary Figure S4.** Unadjusted Kaplan-Meier curves for the Danish data (time 0 = 365 days, i.e., 1 year of age; unit on x-axis = years). Number of children at risk is shown below the graph. p-values from the proportional hazard assumption test for exposure are shown. The p-values were tested with significant level alpha=0.05. The proportionality assumption does not fail if the p-values is greater than the significant level.

**A) Long-term opioid analgesics exposed vs unexposed (“opioid discontinuers”)**

**
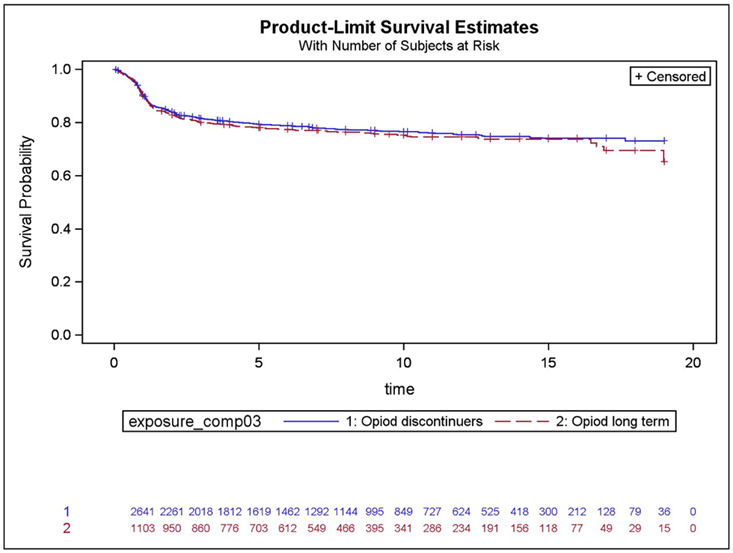
**

**p = 0.8276**

**B) Long-term opioid analgesics exposed vs short-term exposed**

**
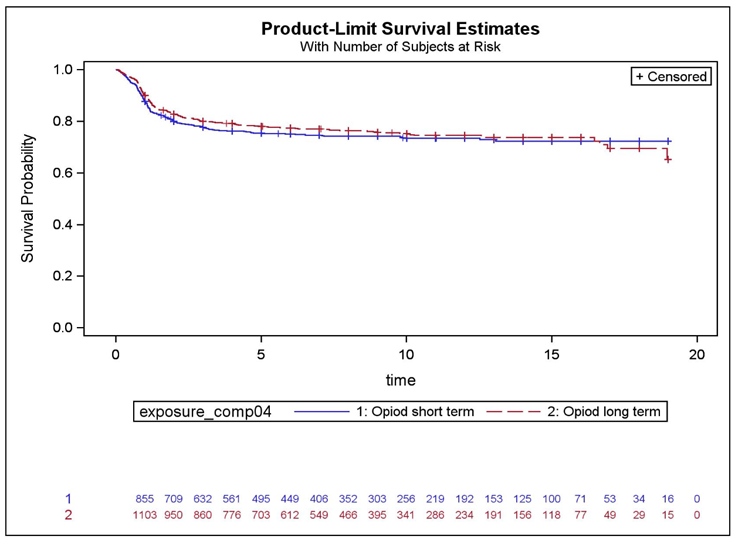
**

**p = 0.046 (~0.050)**

**C) OMT exposed vs OMT unexposed**

**
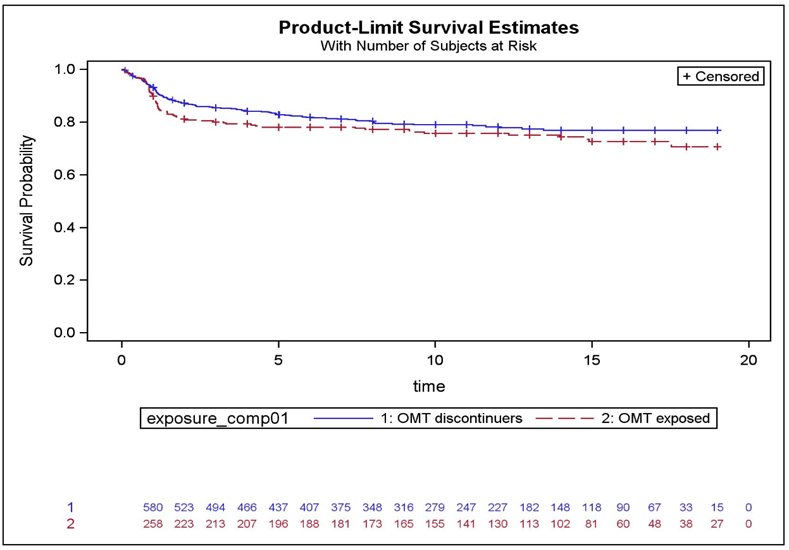
**

**p = 0.222**


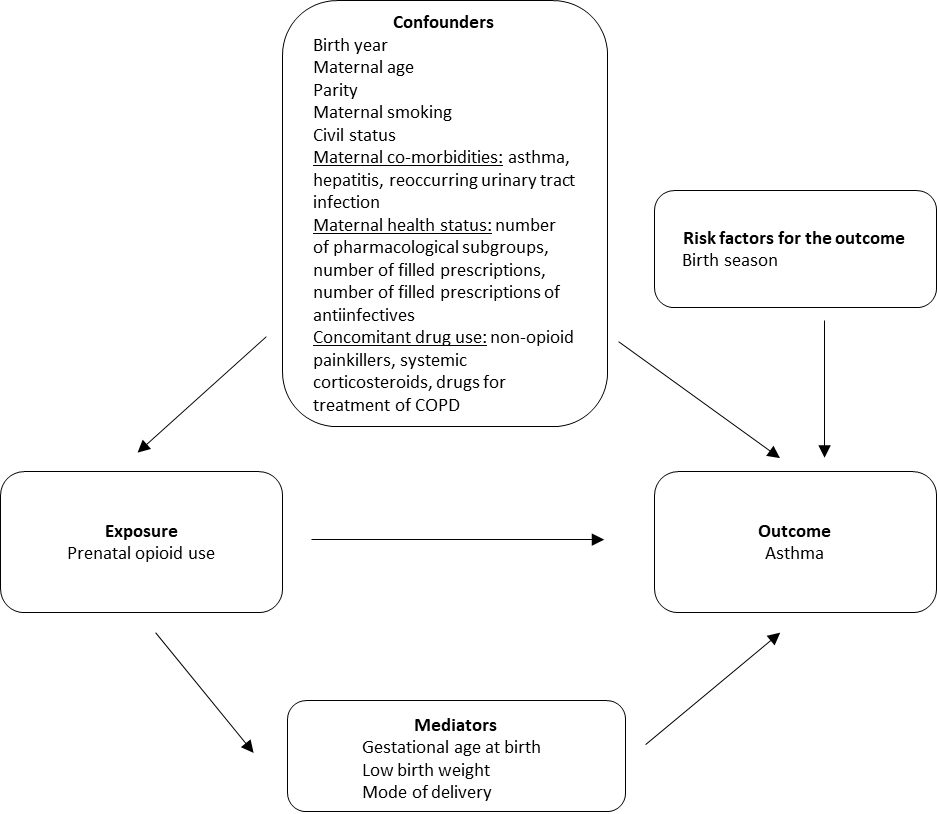


**Supplementary Figure S5**. Directed acyclic graph showing covariates considered as confounders or risk factors for the outcome. These covariates were included in the propensity score. Gestational age at birth, low birth weight and mode of delivery were considered mediators and not included in the propensity score.
